# Identification of prognostic aging-related genes associated with immunosuppression and inflammation in head and neck squamous cell carcinoma

**DOI:** 10.18632/aging.104199

**Published:** 2020-11-24

**Authors:** Jing Yang, Qingshan Jiang, Lijun Liu, Hong Peng, Yaya Wang, Shuyan Li, Yanhua Tang, Jing Yu, Runliang Gan, Zhifeng Liu

**Affiliations:** 1 Department of Gastroenterology, The First Affiliated Hospital of University of South China, Hengyang 421001, Hunan Province, P.R. China; 2 Cancer Research Institute, Hunan Province Key Laboratory of Tumor Cellular and Molecular Pathology, University of South China, Hengyang 421001, Hunan Province, P.R. China; 3 Department of Otorhinolaryngology, The First Affiliated Hospital of University of South China, Hengyang 421001, Hunan Province, P.R. China

**Keywords:** head and neck squamous cell carcinoma (HNSCC), aging-related genes (AGs), prognosis, inflammation, immunosuppression

## Abstract

Aging is regarded as a dominant risk factor for cancer. Additionally, inflammation and asthenic immune surveillance with aging may facilitate tumor formation and development. However, few studies have comprehensively analyzed the relationship between aging-related genes (AGs) and the prognosis, inflammation and tumor immunity of head and neck squamous cell carcinoma (HNSCC). Here, we initially screened 41 differentially expressed AGs from The Cancer Genome Atlas (TCGA) database. In the training set, a prognosis risk model with seven AGs (APP, CDKN2A, EGFR, HSPD1, IL2RG, PLAU and VEGFA) was constructed and validated in the TCGA test set and the GEO set (*P* < 0.05). Using univariate and multivariate Cox regression analyses, we confirmed that risk score was an independent prognostic factor of HNSCC patients. In addition, a high risk score was significantly correlated with immunosuppression, and high expression of PLAU, APP and EGFR was the main factor. Furthermore, we confirmed that a high risk score was significantly associated with levels of proinflammatory factors (IL-1α, IL-1β, IL-6 and IL-8) in HNSCC samples. Thus, this risk model may serve as a prognostic signature and provide clues for individualized immunotherapy for HNSCC patients.

## INTRODUCTION

Head and neck squamous cell carcinoma (HNSCC) ranks sixth among cancer-related deaths, with over 300,000 deaths and 600,000 new cases annually worldwide [1]. The main causes of HNSCC include alcohol consumption, smoking, and high-risk human papillomavirus (HR-HPV) infection [2, 3]. Despite innovations in the multimodal treatment of HNSCC, including surgery, chemoradiotherapy, and targeted drugs, the 5-year survival rate has not improved significantly [4]. Therefore, robust and reliable biomarkers are necessary for efficient early diagnosis and individualized intervention strategies to decrease the mortality rates of HNSCC patients.

Aging is regarded as a time-based or progressive decline of internal physiological function and as a dominant risk factor for many chronic diseases, including cancer, which is now a hot field of cancer research [5, 6]. Cellular senescence acts as a key contributor to the aging progress and to the development of cancers [7]. The effects of senescent cells on tumors are extremely complex, which can be both profitable and deleterious. The senescent neoplastic cells induced by oncogenesis can cause cell-cycle arrest, which appears to be a puissant anti-tumor mechanism [8]. However, the effect of senescent cells on neighboring cancer cells may be the opposite and is closely associated with the secretion of senescence-associated secretory phenotype (SASP) factors [9–11]. Furthermore, cellular senescence-related inflammation has dual effects on tumor immunity [12–14]. Aging-related genes (AGs) play an important role in the regulation of cellular senescence, which can not only inhibit tumors by regulating tumor cell senescence but also promote the development, invasion, metastasis, progression and poor prognosis of tumor [6, 15–17]. However, few studies have systematically analyzed the relationship between AGs and the prognosis of head and neck squamous cell carcinoma (HNSCC). In addition, their correlations with inflammation and tumor immunity in HNSCC remain unclear.

The human aging genome resource (HAGR) is a database identifying a robust set of aging-specific network characteristics that has revealed aging-related genes as network hubs via systemic analysis of the biology and genetics of the human aging process [18]. To evaluate the prognostic values of AGs in HNSCC, we downloaded gene expression profiles of HNSCC patients from The Cancer Genome Atlas (TCGA) database for risk model construction to reveal the AG set related with the prognosis of HNSCC and the potential association between the risk model and inflammation and tumor immunity.

## RESULTS

### Analysis of differentially expressed AGs in HNSCC samples

**Figure 1:**
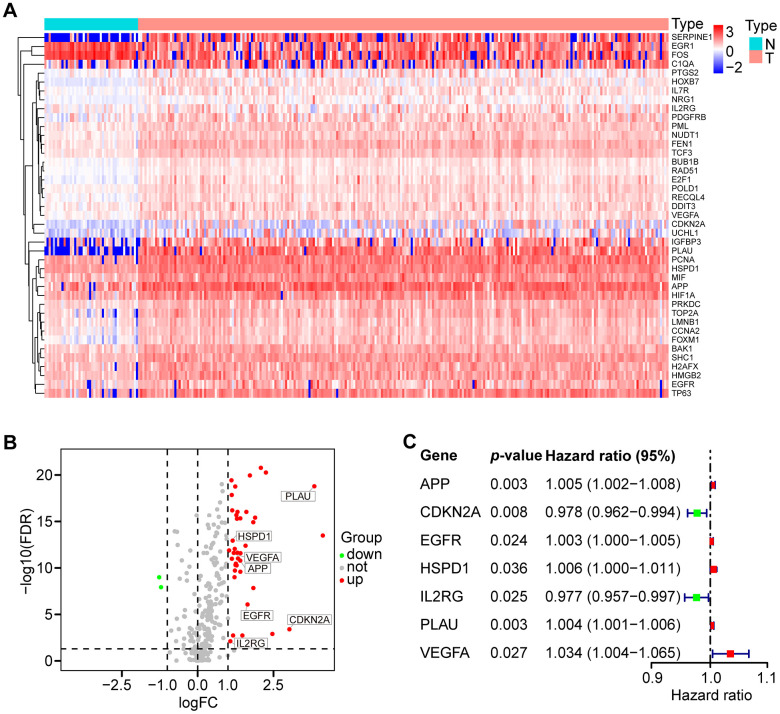
**Differential expression of aging-related genes (AGs) and 7 AGs of prognostic risk models in HNSCC samples.** (**A**) Hierarchical cluster heat map visualizing 41 differentially expressed AGs (DEAGs). (**B**) Volcano plot showing 39 upregulated and 2 downregulated DEAGs (FDR < 0.05 and |logFC| > 1). (**C**) Forest plot showing the characteristics of 7 risk DEAGs in the prognostic risk models.

A total of 307 human AGs (Supplementary Table 1) obtained from the HAGR were distributed on all chromosomes, except for sex chromosome Y (Supplementary Figure 1). Based on the expression of the AGs in the TCGA dataset (clinical characteristics are showed in Table 1), we identified 41 differentially expressed AGs (DEAGs), including 39 upregulated and 2 downregulated DEAGs (FDR < 0.05 and |logFC| > 1). The DEAGs are listed in Supplementary Table 2 and are visualized with a hierarchical cluster heat map (Figure 1A) and a volcano plot (Figure 1B).

**Table 1:** Clinical characteristics of HNSCC patients in the TCGA and GEO data sets.

**Clinical characteristics**	**TCGA**		**GEO (GSE65858)**	
	**n=500**	**%**	**n=270**	**%**
**Age**				
< 60	220	44.0	153	56.7
≥ 60	280	56.0	117	43.3
**Gender**				
Female	133	26.6	47	17.4
Male	367	73.4	223	82.6
**Histologic grade**				
G1-2	360	72.0		
G3-4	121	24.2		
Gx	16	3.2		
**NA**	3	0.6		
Stage				
I-II	114	22.8	55	20.4
III-IV	372	74.4	215	79.6
NA	14	2.8		
**T classification**				
T1-2	176	35.2	115	42.6
T3-4	309	61.8	155	57.4
Tx	11	2.2		
NA	4	0.8		
**N classification**				
N0	239	47.8	94	34.8
N+	239	47.8	176	65.2
Nx	18	3.6		
NA	4	0.8		
**M classification**				
M0	470	94.0	263	97.4
M1	5	1.0	7	2.6
Mx	20	4.0		
NA	5	1.0		
**Vital status**				
Deceased	218	43.6	94	34.8
Living	282	56.4	176	65.2

### Functional analysis of DEAGs in the TCGA data set

**Figure 2:**
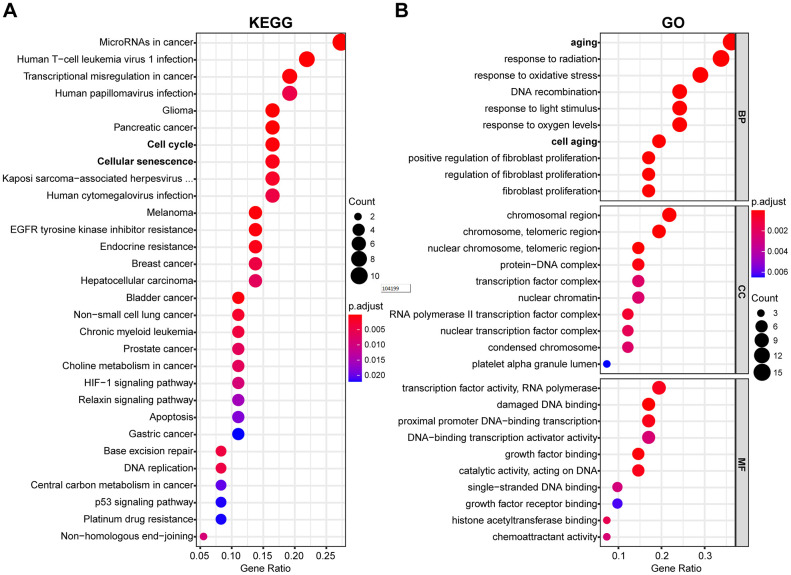
**Functional enrichment analysis of DEAGs of the TCGA data set.** (**A**) The top 30 enriched pathways from KEGG pathway analysis are displayed using an enriched scatter diagram. (**B**) The top 10 enrichment GO terms of BP, CC and MF for DEAGs are also displayed with a scatter diagram. KEGG, Kyoto Encyclopedia of Genes and Genomes. GO, Gene Ontology; BP, biological process; CC, cell component; MF, molecular function.

The potential function and connection of DEAGs in the TCGA dataset were analyzed using GO and KEGG pathway analyses. The top 30 enriched pathways of KEGG pathway analysis are shown as an enriched scatter diagram (Figure 2A). These results revealed that the DEAGs might be associated with cell cycle, cellular senescence, microRNAs in cancer, human T-cell leukemia virus 1 infection and other KEGG pathways in multiple cancers. The top 10 enriched GO terms of BP, CC and MF for the DEAGs are also shown as a scatter diagram (Figure 2B). The most significantly enriched term in the biological process was related to the aging process. These GO terms were also associated with the occurrence and development of cancer. The functional analysis revealed that the DEAGs are closely related to aging, cellular senescence and cancer.

### Identification of a prognostic risk model in the TCGA training set

To identify prognostic DEAGs in HNSCC, the expression of the 41 DEAGs from the TCGA training set was assessed by univariate Cox regression analysis. Seven survival-associated DEAGs, including APP, CDKN2A, EGFR, HSPD1, IL2RG, PLAU and VEGFA, in HNSCC are shown by a forest plot (Figure 1C). Then, a prognostic risk model of 7 survival-associated DEAGs was constructed with LASSO regression analysis. The information and the coefficient values of the 7 genes are displayed in Table 2. The prognostic risk score of each patient was calculated with the following formula:

**Table 2:** The seven genes associated with the risk model in HNSCC.

**ENSG ID**	**Symbol**	**Location**	**Expression status**	**Coefficient**
ENSG00000142192	APP	Chromosome 21	Upregulated	0.0026
ENSG00000147889	CDKN2A	Chromosome 9	Upregulated	-0.0132
ENSG00000146648	EGFR	Chromosome 7	Upregulated	0.0016
ENSG00000144381	HSPD1	Chromosome 2	Upregulated	0.0048
ENSG00000147168	IL2RG	Chromosome X	Upregulated	-0.0113
ENSG00000122861	PLAU	Chromosome 10	Upregulated	0.0017
ENSG00000112715	VEGFA	Chromosome 6	Upregulated	0.0215

Risk score = APP * 0.0026 + CDKN2A * (-0.0132) + EGFR * 0.0016 + HSPD1 * 0.0048 + IL2RG * (-0.0113) + PLAU * 0.0017 + VEGFA * 0.0215

**Figure 3:**
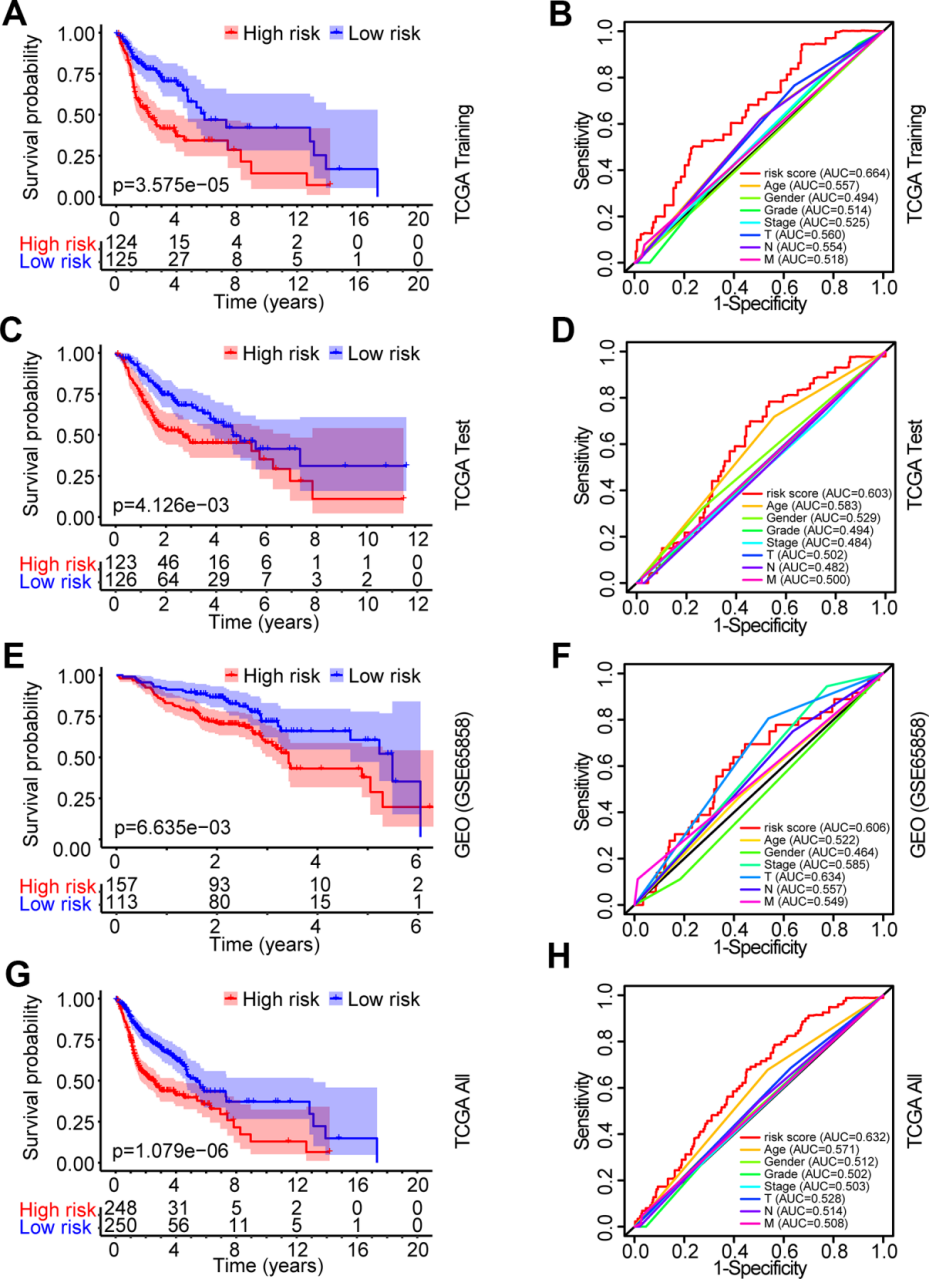
**Identification and verification of the prognostic risk model in HNSCC.** (**A**) Kaplan-Meier survival curve analysis of OS in the high-risk and low-risk groups of HNSCC patients in the TCGA training set. (**B**) ROC curve analysis and AUC for the risk score of AGs in the TCGA training set. (**C**, **E**, **G**) Kaplan-Meier survival curve analysis of OS in the high-risk and low-risk groups of HNSCC patients in the TCGA test set, GEO data set and TCGA all data set, respectively. (**D**, **F**, **H**) ROC curve analysis and AUC for the risk score of AGs in the TCGA test set, GEO data set and TCGA all data set, respectively.

**Figure 4:**
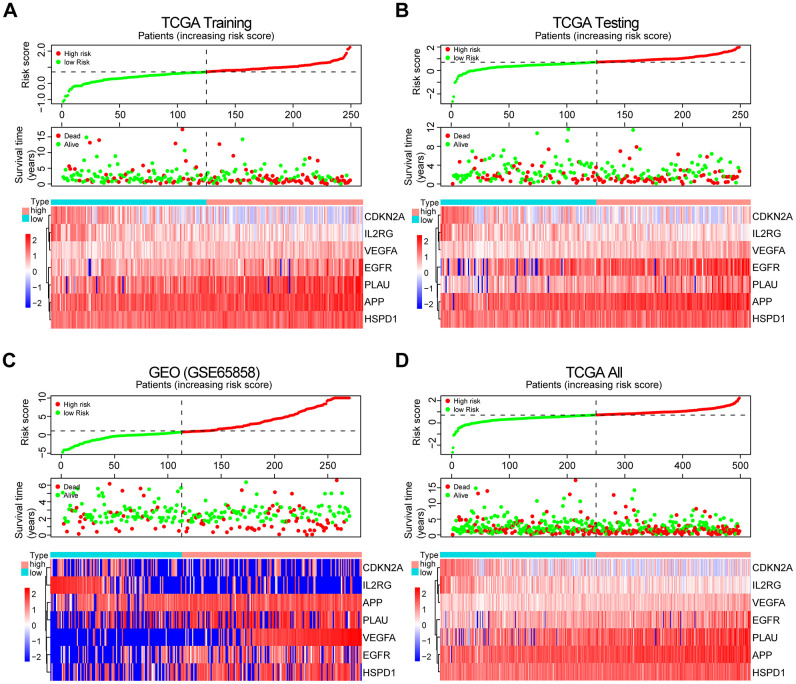
**Prognosis and expression of risk genes in the high-risk and low-risk groups of HNSCC patients.** (**A**) Risk plot distribution, survival status, and expression of risk genes of HNSCC patients in the TCGA training set. (**B**) Risk plot distribution, survival status, and expression of risk genes of HNSCC patients in the TCGA test set. (**C**) Risk plot distribution, survival status, and expression of risk genes of HNSCC patients in the GEO test set. (**D**) Risk plot distribution, survival status, and expression of risk genes of HNSCC patients in the TCGA all data set.

The patients from the TCGA training set were classified into low-risk and high-risk groups based on the median cutoff value of the risk score (0.709). Survival analysis indicated that the overall survival (OS) of the high-risk group was significantly worse than that of the low-risk group (*P* < 0.001, Figure 3A). The receiver operating characteristic (ROC) curves analysis demonstrated acceptable discrimination with the area under the ROC (AUC) of 0.664, which was higher than the AUC of other clinical parameters (Figure 3B). The risk plot with the distribution of patients based on risk scores, the survival status of individual HNSCC patients and the heat maps of the expression profiles of the risk genes are displayed in Figure 4A.

### Verification of the prognostic risk model in the validation data sets

To validate the robustness of the prognostic risk model of the risk genes, we tested the model with independent validation data sets. With the prognostic risk model from the TCGA training data set, all patients in the TCGA test data set were also classified into high-risk (n = 123) and low-risk (n = 126) groups. Kaplan-Meier survival curve analysis demonstrated patient OS in the low-risk group was better than that in the high-risk group (*P* < 0.01, Figure 3C). ROC curves analysis for the TCGA test set achieved an AUC of 0.608, which was higher than the AUC for the other clinical parameters (Figure 3D).

Further validation of the risk model for prognostic prediction was performed using an external independent GEO data set (GSE65858) with 270 HNSCC patients (clinical characteristics are showed in Table 1). With the same risk model, the patients in the GEO test set were segregated into high-risk (n = 157) and low-risk (n = 113) groups, and the model could distinguish patient survival between the high-risk and low-risk groups (*P* < 0.01, Figure 3E). The ROC curves analysis for the GEO test set achieved an AUC of 0.606, which was higher than the AUC of the other clinical parameters, except for T stage (Figure 3F).

A similar analysis was also exploited in the TCGA all data set. The patients in the TCGA all set were divided into high-risk (n = 248) and low-risk (n = 250) groups with significantly different survival (*P* < 0.001, Figure 3G). ROC curves analysis for the TCGA all data set achieved an AUC of 0.632, which was higher than the AUC of the other clinical parameters (Figure 3H).

The risk plot distribution, survival status, and expression of risk genes of the patients in the TCGA and GEO (GSE65858) test sets and TCGA all data set are shown in Figure 4B–4D.

### Independent prognostic predictive value of the risk score in HNSCC patients

**Figure 5:**
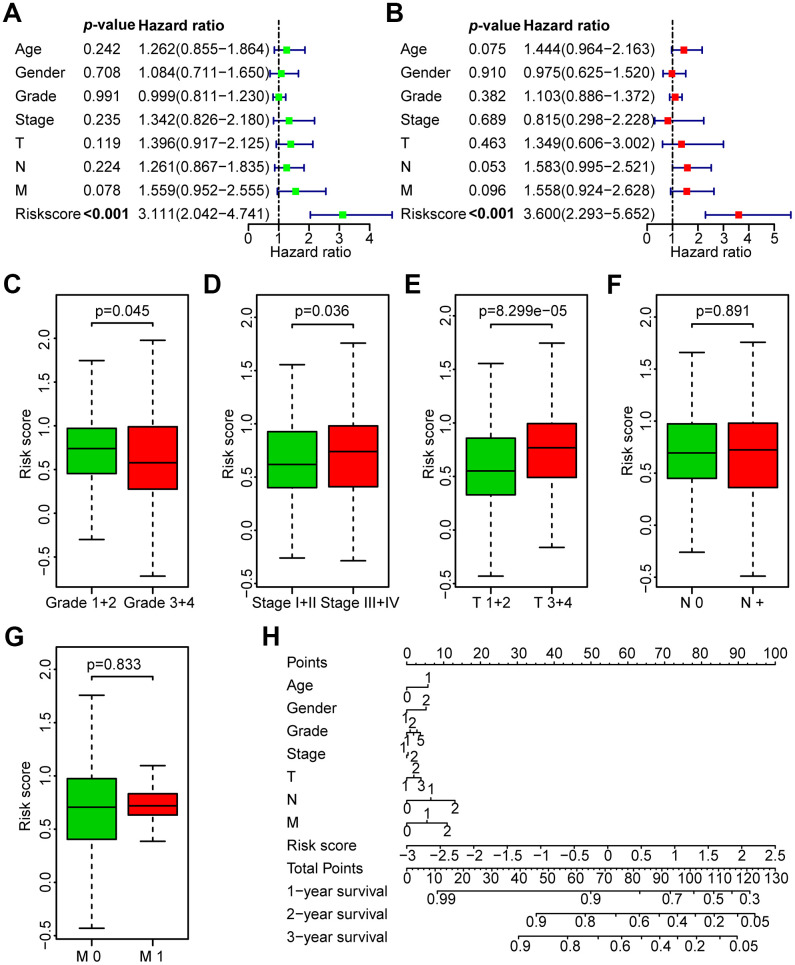
**Correlation between the risk score and the clinicopathological characteristics of HNSCC patients.** (**A**) Univariate Cox regression analysis of clinicopathological parameters of the patients in the TCGA training set. (**B**) Multivariate Cox regression analysis of the clinicopathological parameters of the patients in the TCGA training set. (**C**) Subgroup analysis of pathology grade (Grades 1+2 vs. Grades 3+4). (**D**) Subgroup analysis of clinical stage (Stages I+II vs. Stages III+IV). (**E**) Subgroup analysis of T classification (T 1+2 vs. T 3+4). (**F**) Subgroup analysis of N classification (N0 vs. N+). (**G**) Subgroup analysis of M classification (M 0 vs. M 1). (**H**) Nomogram for OS of HNSCC patients.

To investigate the independence of the risk model in clinicopathological factors, we performed univariate and multivariate Cox regression analysis of the clinicopathological parameters of the patients in the TCGA training set, TCGA test set, GEO test set and TCGA all data set, including risk score, age, gender, grade, clinical stage, and TNM stage. Among the training set and multiple test sets, univariate and multivariate Cox regression analyses showed that only the risk score was consistently significantly associated with prognosis, such as the TCGA training set (HR = 3.111, 95% CI = 2.042-4.741, *P* < 0.001; HR = 3.600, 95% CI = 2.293-5.652, *P* < 0.001, respectively, Figure 5A, 5B), the TCGA test set (HR = 1.573, 95% CI = 1.068-2.316, *P* < 0.05; HR = 1.520, 95% CI = 1.027-2.251, *P* < 0.05, respectively, Supplementary Figure 2A, 2B), the GEO data set (HR = 1.239, 95% CI =1.052-1.459, *P* < 0.05; HR = 1.238, 95% CI = 1.045-1.466, *P* < 0.05, respectively, Supplementary Figure 2C, 2D) and the TCGA all data set (HR = 2.148, 95% CI = 1.619-2.850, *P* < 0.05; HR = 2.202, 95% CI = 1.650-2.939, *P* < 0.05, respectively, Supplementary Figure 2E, 2F). The results revealed that the risk model is a robust prognostic index independent of other clinicopathological parameters.

### Association between the risk score and the clinicopathological characteristics of HNSCC patients in the TCGA data set

Next, the association between the risk score and clinicopathological parameters was investigated (Figure 5C–5G, Table 3). The level of risk score was significantly related to histological grade (*P* < 0.05), clinical stage (*P* < 0.05), and T stage (*P* < 0.001). However, it was not correlated with other clinicopathological parameters, including N stage (*P* = 0.891) or M stage (*P* = 0.833). These results demonstrate that the risk score is closely associated with the progression of HNSCC. Next, a nomogram containing the risk score and clinicopathological parameters was constructed (Figure 5H).

**Table 3:** Correlation between the clinicopathologic characteristics and the risk score (logistic regression) in HNSCC patients in the TCGA data set.

**Clinical characteristics**	**Total (N)**	**Odds ratio in the risk score**	***p*-value**
Age (≥60 vs. <60)	498	1.016(0.713-1.448)	0.928
Gender	498	1.085 (0.729-1.619)	0.685
Grade (G1-2 vs. G3-4)	479	0.630 (0.413-0.953)	0.030
Stage (I-II vs. III-IV)	484	1.484 (0.972-2.278)	**0.039**
Local invasion (T1-2 vs. 3-4)	483	2.173 (1.490-3.188)	**0.000**
Lymph nodes (N0 vs. N+)	476	1.070 (0.747-1.533)	0.714
Distant metastasis (M0 vs. M1)	473	1.513 (0.248-11.564)	0.652

### GSEA of risk score-related signaling pathways

**Figure 6:**
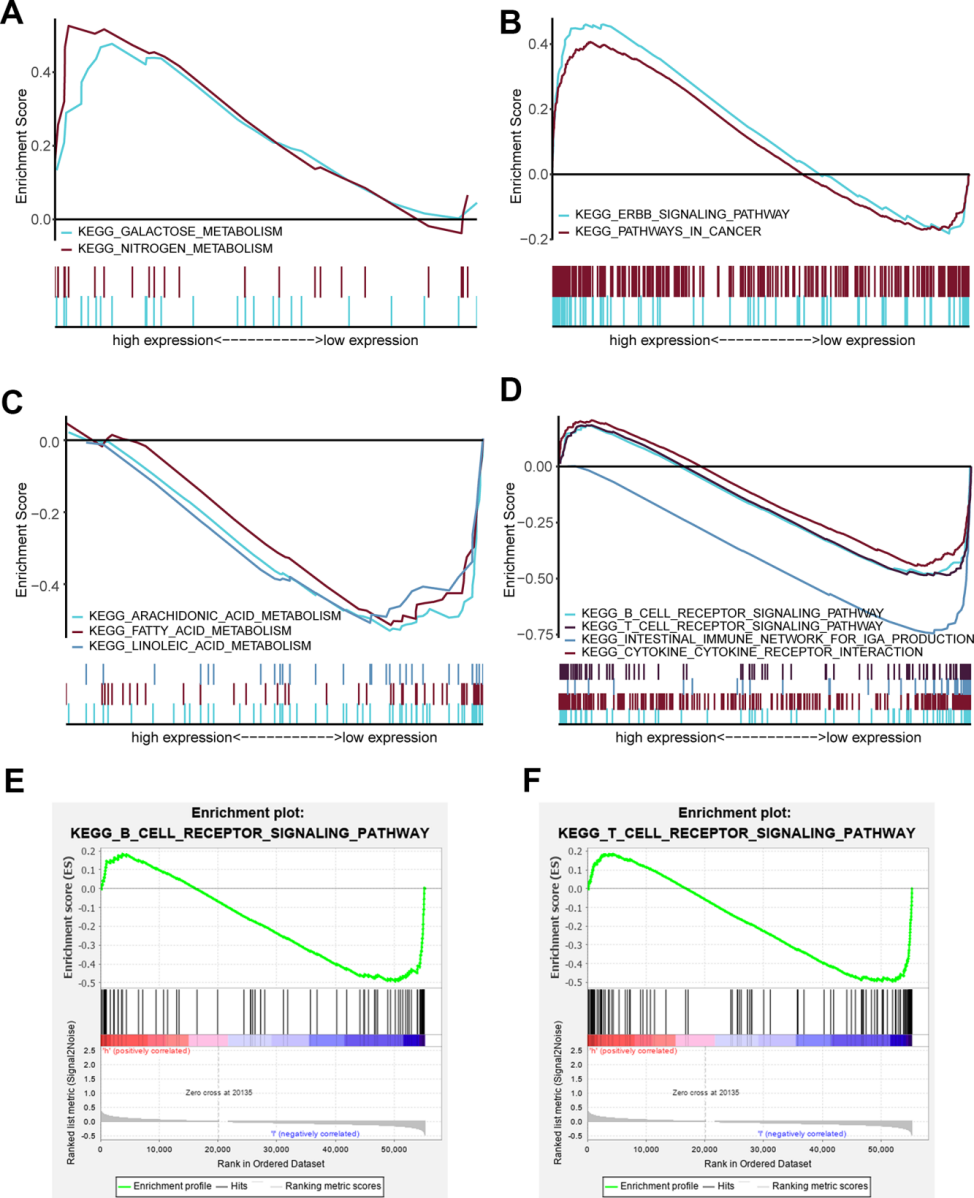
**Enriched pathways of the high-risk and low-risk groups via GSEA.** (**A**) Multiple GSEA for metabolism of the high-risk group: galactose metabolism and nitrogen metabolism. (**B**) Multiple GSEA for cancer pathways of the high-risk group: the ERBB signaling pathway and pathway in cancer. (**C**) Multiple GSEA for metabolism of the low-risk group: arachidonic acid metabolism, fatty acid metabolism and linoleic acid metabolism. (**D**) Multiple GSEA for inflammation and immunity of the low-risk group: the B cell receptor signaling pathway, T cell receptor signaling pathway, intestinal immune network for IgA production and cytokine_cytokine receptor interaction. (**E**) Single GSEA showing the B cell receptor signaling pathway. (**F**) Single GSEA showing the T cell receptor signaling pathway.

GSEA was performed to identify significantly enriched pathways in the high-risk and low-risk groups in the TCGA data set. Thirty enriched pathways in the high-risk and low-risk groups were evaluated (Supplementary Table 5). Enriched pathways with significant differences (FDR < 0.25, NOM *p* < 0.05) were selected (Table 4). The results demonstrated that galactose metabolism, nitrogen metabolism, ERBB signaling pathway and pathways in cancer were significantly enriched in the high-risk group (Figure 6A, 6B) and that arachidonic acid metabolism, fatty acid metabolism, linoleic acid metabolism, B cell receptor signaling pathway, T cell receptor signaling pathway, intestinal immune network for IgA production and cytokine_cytokine receptor interaction were significantly enriched in the low-risk group (Figure 6C, 6D). Interestingly, the B cell receptor signaling pathway and T cell receptor signaling pathway were enriched in the low-risk group (Figure 6E, 6F), which indicated that a high risk score may be associated with immunosuppression. Other individual GSEA diagrams are shown in Supplementary Figure 3.

**Table 4:** Gene sets enriched in high and low risk scores.

**MSigDB collection**	**Name**	**NES**	**ES**	**NOM *p*-value**	**FDR *q*-value**
c2.cp.kegg.v7.1.symbols.gmt	KEGG_ERBB_SIGNALING_PATHWAY	1.658	0.460	0.024	0.197
	KEGG_PATHWAYS_IN_CANCER	1.619	0.406	0.022	0.193
	KEGG_GALACTOSE_METABOLISM	1.520	0.477	0.042	0.247
	KEGG_NITROGEN_METABOLISM	1.511	0.526	0.044	0.233
KEGG_INTESTINAL_IMMUNE_NETWORK_FOR_IGA_PRODUCTION	-2.047	-0.761	0.000	0.016
	KEGG_ARACHIDONIC_ACID_METABOLISM	-1.916	-0.542	0.002	0.040
	KEGG_FATTY_ACID_METABOLISM	-1.668	-0.531	0.024	0.135
	KEGG_CYTOKINE_CYTOKINE_RECEPTOR_INTERACTION	-1.655	-0.446	0.033	0.139
	KEGG_T_CELL_RECEPTOR_SIGNALING_PATHWAY	-1.633	-0.494	0.042	0.126
	KEGG_B_CELL_RECEPTOR_SIGNALING_PATHWAY	-1.594	-0.491	0.048	0.128
	KEGG_LINOLEIC_ACID_METABOLISM	-1.593	-0.533	0.045	0.124

### Association between the risk score and tumor immunity

**Figure 7:**
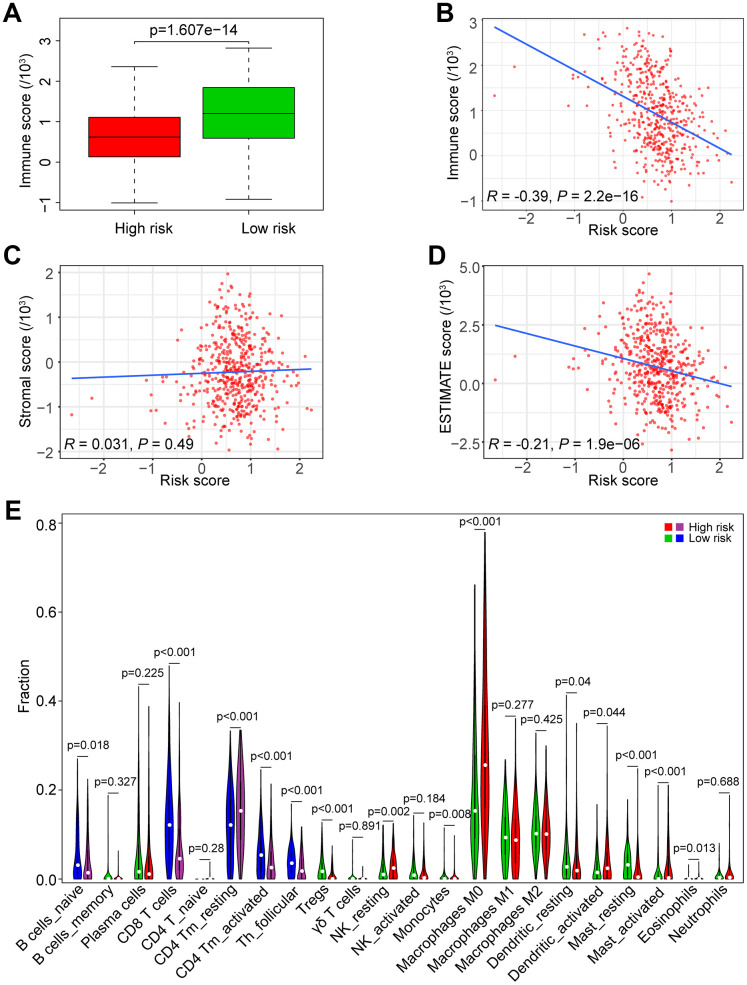
**Association of the risk score with tumor immunity in the TCGA data set.** (**A**) Distribution of immune scores according to the risk score of HNSCC patients. (**B**) Correlation of the risk score with the immune score in HNSCC samples. (**C**) Correlation of the risk score with the stromal score in HNSCC samples. (**D**) Correlation of the risk score with the ESTIMATE score in HNSCC samples. (**E**) Comparisons of immune cells between the high-risk and low-risk groups.

To investigate the association between the risk score and immune/stromal score, we used the ESTIMATE algorithm to evaluate the immune/stromal score of the TCGA data set. The low-risk group had higher immune scores in tumor samples than the high-risk group (*P* < 0.0001, Figure 7A). In addition, Spearman’s rank test revealed that there was a significant negative correlation between the risk score and immune score in HNSCC samples (*R* = -0.39, *P* < 0.0001, Figure 7B). However, there was no significant correlation between the risk score and stromal score in HNSCC samples (*R* = 0.031, *P* = 0.031, Figure 7C). The correlation between the risk score and ESTIMATE score in HNSCC samples was also tested (*R* = -0.21, *P* < 0.0001, Figure 7D).

We estimated the composition fraction of tumor-infiltrating immune cell types of patients in the TCGA data set utilizing the CIBERSORT algorithm to compare the relationship between the risk score and immune cells (Figure 7E). The results showed that HNSCC samples in the high-risk group contained a lower fraction of naïve B cells (*P* < 0.05), CD8 T cells (*P* < 0.001), CD4 memory activated T cells (*P* < 0.001) and follicular helper T cells (*P* < 0.001) compared to those in the low-risk group. However, CD4 memory resting T cells exhibited the opposite result (*P* < 0.001). These results are consistent with those of the GSEA in Figure 6E, 6F, which showed that a high risk score was associated with immunosuppression.

### Correlation of the five immune cells with genes in the risk model

**Figure 8:**
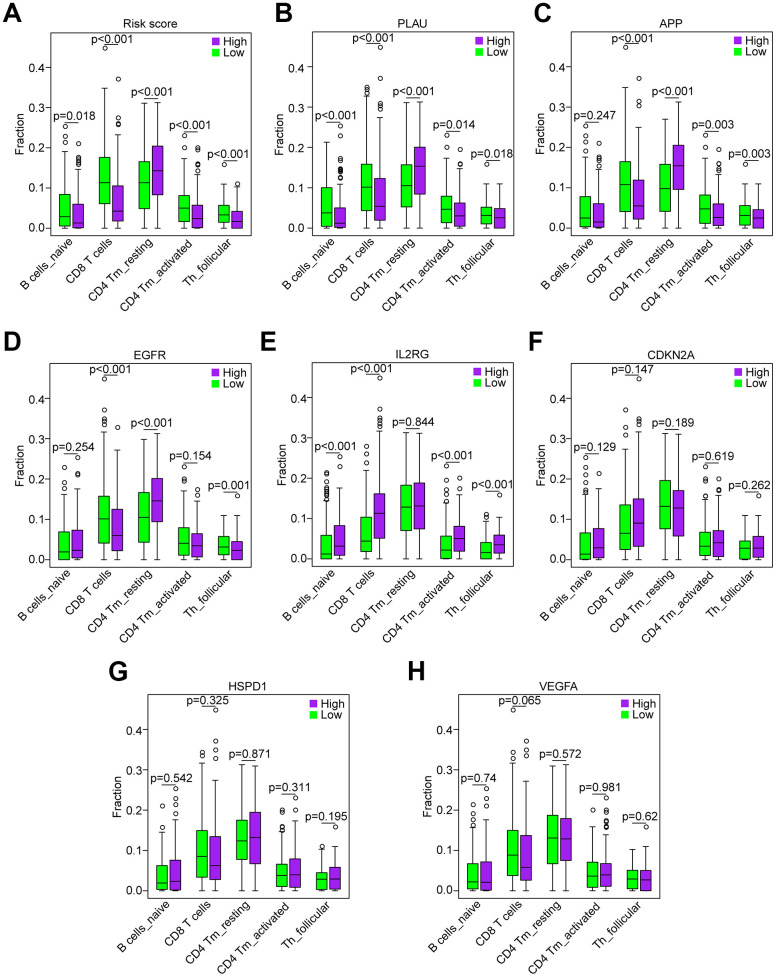
**Correlation of the five immune cells with genes of the risk model in the TCGA data set.** (**A**) Comparison of the five immune cells (naïve B cells, CD8 T cells, CD4 memory activated T cells and follicular helper T cells) between the high-risk and low-risk groups. (**B**–**H**) Distribution of the five immune cells based on the high expression and low expression of PLAU, APP, EGFR, IL2RG, CDKN2A, HSPD1 and VEGFA.

Based on the association between the risk model and the above five immune cell types (Figure 8A), we analyzed the correlation of the five immune cells with the 7 genes of the risk model. HNSCC samples with high PLAU expression contained a lower fraction of naïve B cells, CD8 T cells, CD4 memory activated T cells and follicular helper T cells compared to those with low expression, and CD4 memory resting T cells exhibited the opposite result (all *P* < 0.05, Figure 8B), which was consistent with the risk score. HNSCC samples with high APP expression compared to those with low expression showed that except for naïve B cells (*P* = 0.247), the results were consistent with the result of the risk score (all *P* < 0.01, Figure 8C). Similarly, the results of EGFR, except naïve B cells (*P* = 0.254) and CD4 memory activated T cells (*P* = 0.254), were consistent with the result of the risk score (all *P* < 0.01, Figure 8D). However, the results of IL2RG, as a protective factor, were not consistent with the results of the risk score (Figure 8E). Furthermore, the results of CDKN2A, HSPD1 and VEGFA showed that there was no significant difference in the five immune cells between the patients with high and low expression (all *P* > 0.05, Figure 8F–8H). Hence, the aging-related genes PLAU, APP and EGFR play a key role in immunosuppression of HNSCC.

### Correlation of proinflammatory factors with the risk score and genes of the risk model

**Figure 9:**
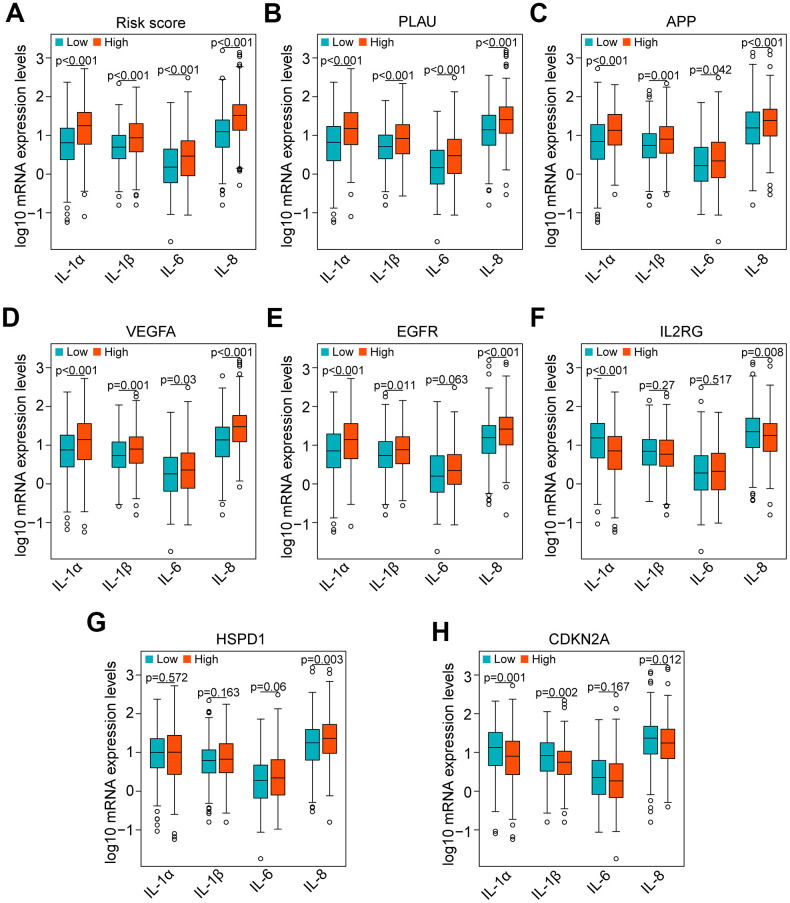
**Correlation of proinflammatory factors with the risk score and genes of the risk model in the TCGA data set.** (**A**) Comparison of the main proinflammatory factors (IL-1α, IL-1β, IL-6 and IL-8) between the high-risk and low-risk groups. (**B**–**H**) Distribution of the main proinflammatory factors based on high and low expression of PLAU, APP, VEGFA, EGFR, IL2RG, HSPD1 and CDKN2A.

Currently, accumulating studies have demonstrated that chronic inflammation related to cellular senescence plays a key role in tumor immunosuppression and major proinflammatory factors, including IL-1α, IL-1β, IL-6 and IL-8 [12–14]. Therefore, we investigated the correlation of major proinflammatory factors with the risk score and genes in the risk model. The results showed that mRNA expression levels of IL-1α, IL-1β, IL-6 and IL-8 in high-risk HNSCC samples were significantly higher than those in low-risk samples (all *P* < 0.001, Figure 9A). Consistent with the result of the risk score, the results of PLAU, APP and VEGFA also showed that the mRNA expression levels of IL-1α, IL-1β, IL-6 and IL-8 in HNSCC samples with high expression of PLAU, APP and VEGFA were significantly higher than those with low expression (all *P* < 0.05, Figure 9B–9D). Similarly, the results of EGFR, except for IL-6 (*P* = 0.063), were consistent with the results of the risk score (all *P* < 0.05, Figure 9E). However, the results of IL2RG, HSPD1 and CDKN2A were either opposite of the results of the risk score, or there was no significant difference (Figure 9F–9H).

## DISCUSSION

One key hallmark of cancer is the ability of cancer cells to evade immune destruction [26]. In the 1960s, studies showed that the aging process was associated with decreased immunological function [27–29]. Subsequently, accumulating studies have demonstrated that a chronic inflammatory microenvironment is involved in the aging process [30, 31]. Inflammation and asthenic immune surveillance with aging may facilitate tumor formation and development [9]. Currently, the role of the aging process in HNSCC is still ambiguous. Therefore, exploring the expression patterns of aging-related genes (AGs) is crucial for understanding the role of the aging process in HNSCC. Few studies have analyzed the correlation of AGs with HNSCC. Furthermore, the correlation of the expression of AGs with the prognosis of HNSCC patients has not been systematically investigated.

In this study, we initially performed a comprehensive investigation of the associations between 41 differentially expressed AGs (DEAGs, Figure 1A, 1B) and HNSCC prognosis and constructed a prognosis risk model with seven AGs, including APP, CDKN2A, EGFR, HSPD1, IL2RG, PLAU and VEGFA (Figure 1C) that revealed a robust performance signature for predicting prognosis compared to clinicopathological factors in training and multiple validation sets (Figure 3).

In the prognostic risk model, CDKN2A and IL2RG acted as protective factors, while APP, PLAU, VEGFA, EGFR and HSPD1 were risk factors (Figure 4). CDKN2A, upregulated by Lupeol, had been shown to cause cell cycle arrest at G1 phase, mediating antitumor effects in HNSCC [32]. Low CDKN2A expression predicted poor survival independently of other clinical factors in HPV-negative HNSCC [33]. IL2RG knockout mice showed immunodeficiency [34] and high tumorigenic engraftment efficiency of human cancer cells and tissues [35]. These studies are consistent with our results, suggesting that CDKN2A and IL2RG are anticancer genes. Previous studies showed that APP, a protein that originates β-amyloid that constitutes amyloid plaques, acts as one of the main pathological features of AD, one of the major aging-related diseases [36]. Interestingly, APP depletion causes cell cycle arrest in breast cancer cells and non-small cell lung cancer [37, 38]. PLAU has been identified as a biomarker of HNSCC [39], but its relationship to inflammation and immunity has not been explored. VEGFA was demonstrated to be involved in age-related macular degeneration (AMD), a leading cause of visual impairment in aging populations [40]. Indeed, VEGFA is a key molecule in various signaling pathways promoting the progression of multiple cancers [41–43]. EGFR is a biomarker in HNSCC [44] and enhances the progression of HNSCC by mediating a variety of signaling pathways [45–47]. HSPD1 was closely related to prognosis in both oral squamous cell carcinoma and breast cancer [48, 49]. Our study was the first to suggest that these factors represent a prognostic risk model of HNSCC.

Interestingly, our GSEA results revealed that B cell receptor (BCR) signaling and T cell receptor (TCR) signaling pathways were enriched in the low-risk group (Figure 6E, 6F), indicating that they may be inhibited in the high-risk group. BCR and TCR signaling is crucial for B cell and T cell proliferation and for development of adaptive immunity, and their abnormalities could lead to immunodeficiency [50–53]. For these reasons, we investigated correlation of the risk score with the immune score and the composition fraction of tumor-infiltrating immune cell types in HNSCC samples of TCGA data set. As we confirm here, there was a negative correlation between the risk score, and the immune and high-risk group contained lower fractions of naïve B cells, CD8 T cells, CD4 memory activated T cells and follicular helper T and a higher fraction of CD4 memory resting T cells compared to the low-risk group (Figure 7). Hence, our study revealed a significant correlation between a high risk score and immunosuppression and its association with the growth and differentiation of B and T cells, and high expression of PLAU, APP and EGFR were the main factors of tumor immunosuppression (Figure 8). Previous work unveiled that PLAU is particularly important for memory regulatory T cells (Tregs) [54]; however, there was no investigation of other immune cells. Based on an Alzheimer's transgenic mouse, PLAU was demonstrated as an impact factor of adaptive immunity, involved in lacking functional B and T cells [55]. The EGFR monoclonal antibody, Cetuximab, triggered immunogenic cell death [56], suggesting that EGFR plays an important role in immune cells survival. The proinflammatory factors of the senescence associated secretory phenotype (SASP) facilitate tumor immunosuppression [30, 31]. We confirmed that a high risk score was significantly associated with mRNA expression levels of IL-1α, IL-1β, IL-6 and IL-8 in HNSCC samples, and the results of PLAU, APP, VEGFA and EGFR were consistent with the results of the risk score (Figure 9).

Although we identified a prognostic risk model with seven AGs and confirmed that the risk model was significantly associated with inflammation and immunosuppression, this work has limitations. The study conducted with bioinformatics analysis was not robust enough and needs to be confirmed via experimental verification. Hence, further laboratory experiments, including a multicenter study with larger sample sizes, are needed.

In summary, in this study, we developed a robust prognostic risk model with 7 AGs. Compared to other clinical parameters, the risk score is an independent prognostic index. Furthermore, a high risk score indicates the chronic inflammatory and immunosuppressive state of HNSCC patients. Therefore, this risk model may serve as a prognostic signature and provide clues for individualized immunotherapy for HNSCC patients.

## CONCLUSIONS

In conclusion, in our study, we developed a prognostic risk model with 7 differentially expressed AGs, which has great potential as an immunosuppressive and inflammatory state biomarker in HNSCC patients and provides insight into individualized immunotherapy for HNSCC patients.

## MATERIALS AND METHODS

### Data sets

We obtained 307 human AGs from the human aging genome resource (HAGR, http://genomics.senescence.info/genes/, Supplementary Table 1). The RNA sequencing (RNA-Seq) expression profile data set was downloaded from the TCGA database (https://portal.gdc.cancer.gov/) of 500 HNSCC patients (2 duplicate patients were removed) with 44 paracarcinoma samples, and clinical information was downloaded from the cBioPortal database (https://www.cbioportal.org/). For TCGA data, 498 HNSCC patients with follow-up data were selected and randomly divided into two groups: the TCGA training set (n=298, Supplementary Table 3) and the TCGA test set (n=298, Supplementary Table 4). The GSE65858 data set as an independent verification set was obtained from the Gene Expression Omnibus (GEO) database (https://www.ncbi.nlm.nih.gov/geo/) and included RNA-Seq data and clinical information. We performed data analysis utilizing R software (version 3.6.3, https://www.r-project.org/).

### Differentially expressed gene (DEG) analysis

We evaluated differentially expressed aging-related genes (DEAGs) between HNSCC and normal samples using the Wilcoxon test in the limma package. Cut-off values were adjusted with the false discovery rate (FDR) [19]. FDR < 0.05, and |log FC| value > 1 was defined as significant. Then, we constructed a hierarchical cluster heat map via the “pheatmap” package and a volcano plot to visualize the results of the DEAGs. The distribution of the DEAGs on chromosomes was visualized via the “OmicCircos” R package [20].

### GO and KEGG pathway analyses

Gene Ontology (GO) and Kyoto Encyclopedia of Genes and Genomes (KEGG) pathway enrichment analyses were implemented with the “enrichplot” R package [21] to analyze the function of the DEAGs. GO terms contained biological process (BP), cellular component (CC) and molecular function (MF). For the analysis results, both *P*-value and false discovery rate (FDR) values < 0.05 were defined as statistically significant.

### Construction of a prognostic gene signature

To identify survival-associated DEAGs, we conducted univariate Cox regression analysis. Candidate prognostic genes were selected with a threshold value of *P* < 0.05. Then, Lasso regression analysis in the TCGA training set was executed, and a multigene prognostic signature was constructed. The individualized risk score was calculated with the regression coefficients of each gene using the following computational formula:

$$Risk\;score\;=\sum_{k=1}^{n}coefficient\;(gene_{k})*Exp_{k}$$

where *n* is the number of the candidate prognostic genes, *gene_k_* is the _*k*_th candidate genes, coefficient is the estimated regression coefficient of genes from the multivariate Cox regression analysis, and *Exp_k_* is the expression value of the _*k*_th candidate genes. Based on the median the risk score of the TCGA training set, the HNSCC patients were divided into high-risk and low-risk groups. The association between the candidate genes and risk scores were shown by a hierarchical cluster heat map, and a nomogram containing the risk score of prognostic AGs and clinicopathological parameters was constructed via the “rms” R package.

### Gene Set Enrichment Analysis

GSEA is a powerful analytical method used for estimating significant differences between two biological conditions to determine specific functional gene sets [22]. In the current study, GSEA was performed using GSEA software (v4.0.3) (https://www.gsea-msigdb.org/gsea/downloads.jsp) with the Molecular Signatures Database (MSigDB) [23] C2 curated gene sets, which generated a list of significantly different gene sets between the high-risk and low-risk groups. Each gene set was permutated 1000 times for each analysis. Gene sets with *p-*value < 0.05 and FDR < 0.25 were considered significantly enriched.

### Evaluation of immune scores and immune cell infiltration

The ESTIMATE (Estimation of Stromal and Immune cells in Malignant Tumor tissues using Expression data) algorithm is a tool developed to evaluate immune and stromal scores in tumor samples. We calculated the immune and stromal ESTIMATE scores based on TCGA gene expression data using the “estimate” R package [24].

CIBERSORT is an analytical method used for characterizing the cellular composition of complex tissues based on gene expression profiles [25]. We conducted an estimation of the composition fraction of tumor-infiltrating immune cell types of each patient using the CIBERSORT algorithm (http://cibersort.stanford.edu/).

### Statistical analysis

All statistical analyses were performed using R-3.6.3. The distribution differences among the variables were analyzed by chi-square test or Fisher’s exact test. The Kaplan-Meier curve with the log-rank test was used to estimate survival analysis. Univariate and multivariate Cox regression analyses were performed to analyze the relationship between given factors and survival in HNSCC patients. ROC curve analysis was used to evaluate the diagnostic value of the risk model. Spearman’s rank correlation test was estimated to assess the correlation between variables. *P* < 0.05 was identified as statistically significant.

### Data Availability Statement

Expression profile datasets for this study can be accessed from The Cancer Genome Atlas (TCGA) (https://portal.gdc.cancer.gov/) and Gene Expression Omnibus (GEO) database (https://www.ncbi.nlm.nih.gov/geo/), and aging-related genes (AGs) can be retrieved from the Human Aging Genome Resources (HAGR, http://genomics.senescence.info/genes/).

## Supplementary Material

Supplementary Figures

Supplementary Table 1

Supplementary Table 2

Supplementary Table 3

Supplementary Table 4

Supplementary Table 5
