# Supplementary material for: Identification of prognostic aging-related genes associated with immunosuppression and inflammation in head and neck squamous cell carcinoma
Source: Aging (Albany NY). 2020 Nov 24;12(24):25778–804. doi: 10.18632/aging.104199 (PMC7803584; doi:10.18632/aging.104199)
Supplement: Supplementary Figures [file aging-12-104199-s001.pdf]

SUPPLEMENTARY FIGURES

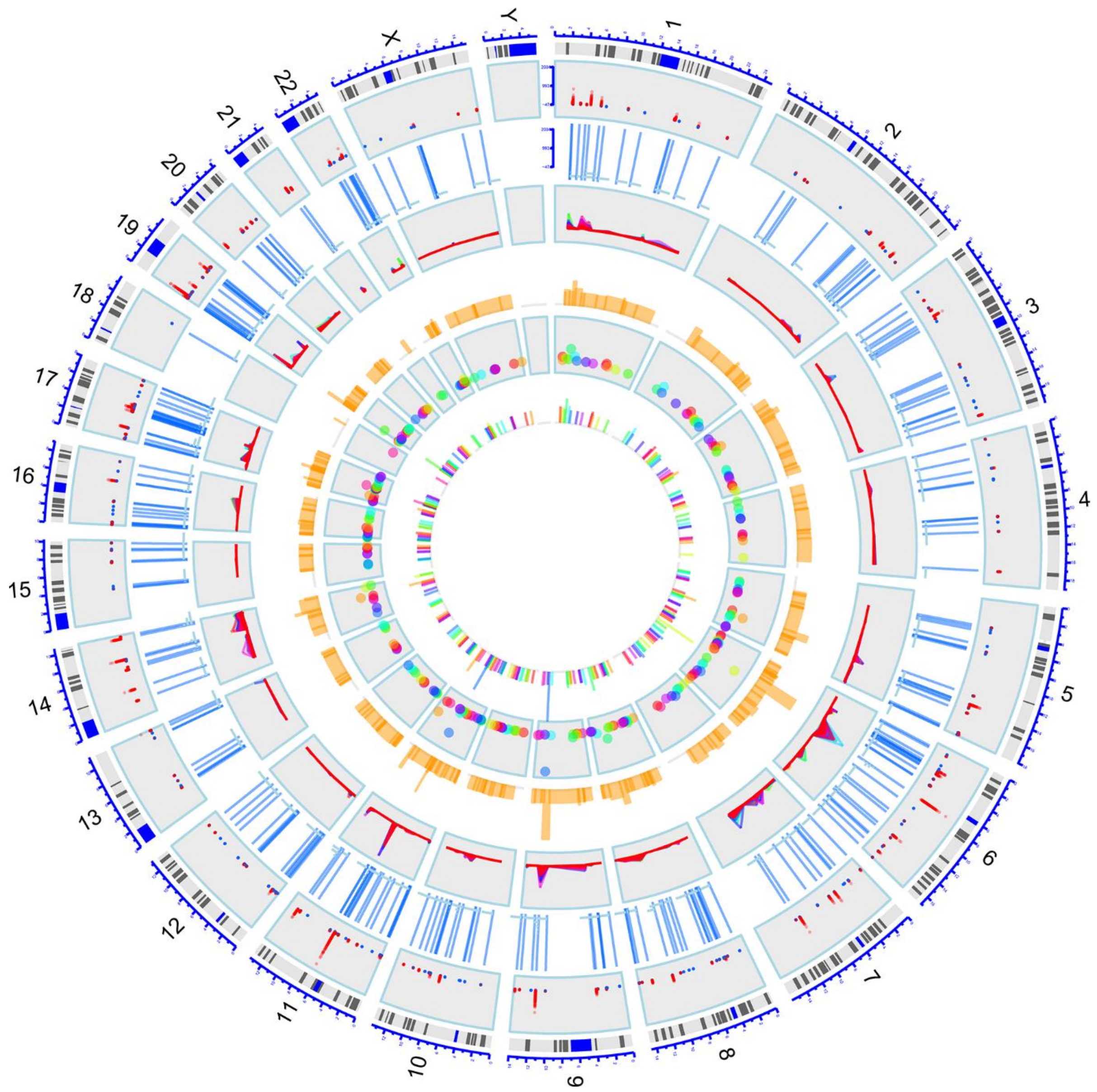

Supplementary Figure 1. Distribution of the DEAGs on chromosomes. A total of 307 human AGs obtained from HAGR were distributing on all chromosomes, except sex chromosome Y.

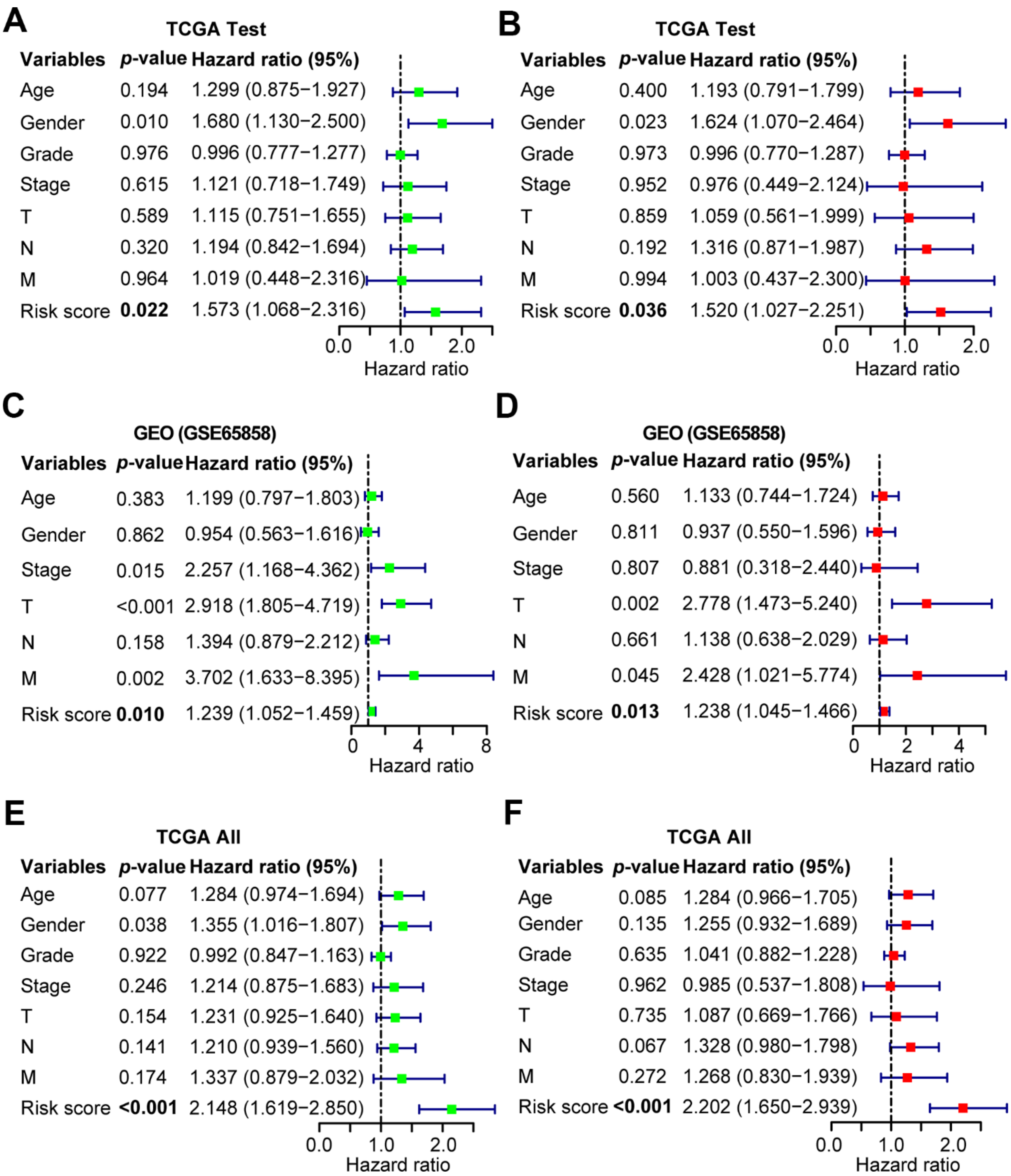

Supplementary Figure 2. Univariate and multivariate cox regression analyses of OS in HNSCC patients with other data sets. (**A**, **B**) Clinicopathological parameters of the patients in the TCGA test set using univariate and multivariate Cox regression analyses, respectively. (**C**, **D**) Clinicopathological parameters of the patients in the GEO (GSE65858) data set using univariate and multivariate Cox regression analyses, respectively. (**E, F**) Clinicopathological parameters of the patients in the TCGA all data set using univariate and multivariate Cox regression analyses.

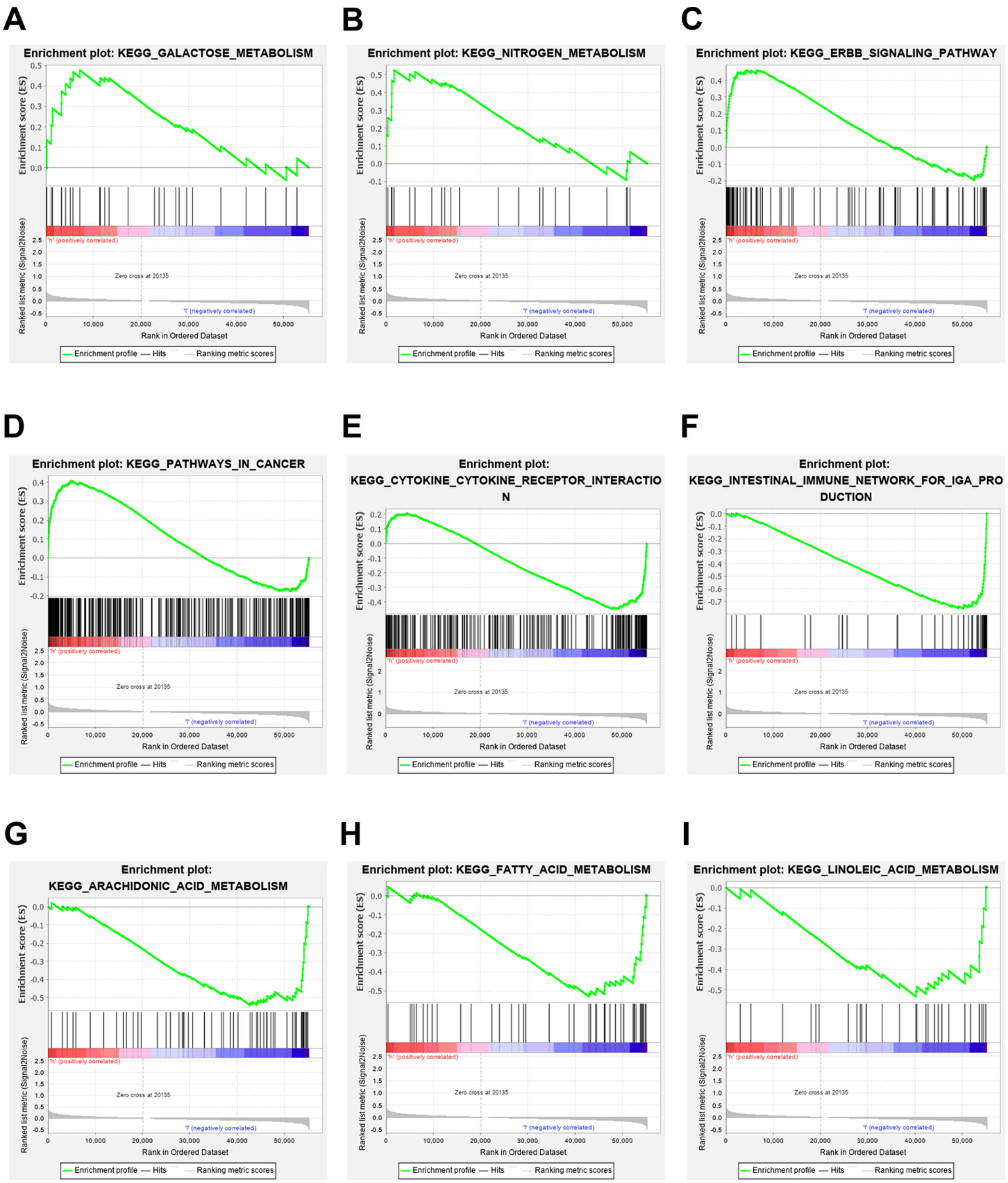

Supplementary Figure 3. Other single GSEA figures in the high-risk and low-risk groups. Single GSEA showing galactose metabolism (**A**), nitrogen metabolism (**B**), the ERBB signaling pathway (**C**), pathway in cancer (**D**), cytokine_cytokine receptor interaction (**E**), intestinal immune network for IgA production (**F**), arachidonic acid metabolism (**G**), fatty acid metabolism (**H**) and linoleic acid metabolism (**I**).
