# Supplementary material for: Identification of prognostic aging-related genes associated with immunosuppression and inflammation in head and neck squamous cell carcinoma
Source: Aging (Albany NY). 2020 Nov 24;12(24):25778–804. doi: 10.18632/aging.104199 (PMC7803584; doi:10.18632/aging.104199)
Supplement: Supplementary Table 2 [file aging-12-104199-s003.pdf]

SUPPLEMENTARY TABLE

Supplementary Table 2. Forty-one differentially expressed genes in the aging-related gene set.

Gene	ConMean	TreatMean	logFC	*p*Value	FDR
APP	39.247603	103.3061	1.3962495	4.95E-13	9.04E-13
BAK1	10.175959	23.5007	1.2075388	1.44E-13	3.18E-13
BUB1B	3.1727858	6.922003	1.1254394	2.82E-18	1.48E-17
C1QA	27.634554	61.46906	1.1533872	0.00116444	0.001223
CCNA2	5.3256053	13.96796	1.391104	3.40E-17	1.10E-16
CDKN2A	1.3581066	10.93876	3.0097805	0.00022727	0.000258
DDIT3	5.1889558	11.92465	1.2004307	2.80E-10	3.66E-10
E2F1	2.4063142	10.09535	2.0687939	6.24E-24	2.62E-22
EGFR	9.4966372	29.37689	1.6291928	3.37E-07	3.93E-07
EGR1	177.67804	76.10728	-1.223159	3.95E-09	4.88E-09
FEN1	6.047823	19.74638	1.7071004	1.23E-22	1.73E-21
FOS	258.19873	106.0324	-1.283977	2.88E-10	3.66E-10
FOXM1	3.1564422	14.86446	2.2354956	3.97E-23	8.34E-22
H2AFX	12.914618	29.94685	1.2133993	4.93E-11	6.90E-11
HIF1A	20.036985	49.84471	1.314775	1.95E-12	3.41E-12
HMGB2	12.80035	33.52158	1.3889068	3.52E-12	5.68E-12
HOXB7	1.9075584	6.730141	1.8189094	9.78E-17	2.74E-16
HSPD1	29.136663	59.11269	1.0206322	2.16E-13	4.54E-13
IGFBP3	14.93991	52.80921	1.821618	4.89E-09	5.87E-09
IL2RG	5.2557188	10.9742	1.0621562	0.00531744	0.005447
IL7R	3.425877	8.258084	1.2693337	1.29E-11	1.86E-11
LMNB1	6.6300363	15.59522	1.234015	1.14E-11	1.71E-11
MIF	17.602226	43.2198	1.2959346	4.06E-13	8.12E-13
NRG1	2.3744663	5.626124	1.244538	7.89E-12	1.23E-11
NUDT1	5.2909544	13.01608	1.2986956	4.57E-18	2.02E-17
PCNA	24.006157	72.29586	1.5905086	4.81E-18	2.02E-17
PDGFRB	5.6199417	14.75284	1.3923654	6.67E-11	9.04E-11
PLAU	5.8109266	82.76741	3.8322227	3.61E-21	3.03E-20
PML	5.9293315	13.05956	1.139165	1.58E-14	3.89E-14
POLD1	4.3692004	9.422398	1.108725	4.68E-20	2.81E-19
PRKDC	8.5063936	19.21432	1.1755628	4.48E-13	8.56E-13
PTGS2	3.8293036	10.52787	1.4590596	0.00116031	0.001223
RAD51	2.9088248	6.234649	1.0998719	5.46E-22	5.73E-21
RECQL4	3.5042193	8.516179	1.2811132	3.91E-17	1.17E-16
SERPINE1	5.2360086	90.50704	4.1114907	3.95E-15	1.04E-14
SHC1	13.929559	33.40669	1.2619875	1.14E-17	4.33E-17
TCF3	6.2120472	14.51215	1.2241211	4.46E-21	3.12E-20

TOP2A	6.0594999	22.28673	1.8789141	2.72E-17	9.52E-17
TP63	18.548258	54.84206	1.563999	6.12E-14	1.43E-13
UCHL1	2.391086	13.05015	2.4483281	0.00077289	0.000854
VEGFA	4.337274	9.386313	1.1137699	2.10E-12	3.53E-12
