# Supplementary material for: Identification of prognostic aging-related genes associated with immunosuppression and inflammation in head and neck squamous cell carcinoma
Source: Aging (Albany NY). 2020 Nov 24;12(24):25778–804. doi: 10.18632/aging.104199 (PMC7803584; doi:10.18632/aging.104199)
Supplement: Supplementary Table 5 [file aging-12-104199-s006.pdf]

## SUPPLEMENTARY TABLE

**Supplementary Table 5. The top thirty enriched pathways of the high and low risk scores.**

| NAME | SIZE | NES | ES | NOM p-val | FDR q-val |
|---|---|---|---|---|---|
| KEGG_FOCAL_ADHESION | 199 | 1.791 | 0.540501 | 0.010 | 0.621 |
| KEGG_WNT_SIGNALING_PATHWAY | 150 | 1.787 | 0.467608 | 0.008 | 0.325 |
| KEGG_PROTEIN_EXPORT | 24 | 1.715 | 0.626685 | 0.023 | 0.391 |
| KEGG_BASAL_TRANSCRIPTION_FACTORS | 35 | 1.677 | 0.565968 | 0.028 | 0.391 |
| KEGG_ADHERENS_JUNCTION | 73 | 1.677 | 0.500147 | 0.036 | 0.313 |
| KEGG_TGF_BETA_SIGNALING_PATHWAY | 85 | 1.674 | 0.492573 | 0.020 | 0.267 |
| KEGG_SMALL_CELL_LUNG_CANCER | 84 | 1.661 | 0.488079 | 0.022 | 0.250 |
| KEGG_ECM_RECEPTOR_INTERACTION | 84 | 1.659 | 0.584678 | 0.047 | 0.221 |
| KEGG_ERBB_SIGNALING_PATHWAY | 87 | 1.658 | 0.460301 | 0.024 | 0.197 |
| KEGG_RENAL_CELL_CARCINOMA | 70 | 1.650 | 0.456580 | 0.028 | 0.189 |
| KEGG_REGULATION_OF_ACTIN_CYTOSKELETON | 213 | 1.625 | 0.422086 | 0.030 | 0.204 |
| KEGG_PATHWAYS_IN_CANCER | 325 | 1.619 | 0.406490 | 0.022 | 0.193 |
| KEGG_GLYCOSAMINOGLYCAN_BIOSYNTHESIS_CHONDROITIN_SULFATE | 22 | 1.618 | 0.620100 | 0.051 | 0.180 |
| KEGG_EPITHELIAL_CELL_SIGNALING_IN_HELICOBACTER_PYLORI_INFECTION | 68 | 1.588 | 0.432624 | 0.028 | 0.201 |
| KEGG_O_GLYCAN_BIOSYNTHESIS | 30 | 1.566 | 0.518195 | 0.047 | 0.214 |
| KEGG_GAP_JUNCTION | 90 | 1.534 | 0.413798 | 0.063 | 0.242 |
| KEGG_GALACTOSE_METABOLISM | 26 | 1.520 | 0.476869 | 0.042 | 0.247 |
| KEGG_UBIQUITIN_MEDIATED_PROTEOLYSIS | 134 | 1.512 | 0.414400 | 0.065 | 0.245 |
| KEGG_NITROGEN_METABOLISM | 23 | 1.511 | 0.525666 | 0.044 | 0.233 |
| KEGG_RNA_DEGRADATION | 59 | 1.506 | 0.465151 | 0.102 | 0.228 |
| KEGG_BASAL_CELL_CARCINOMA | 55 | 1.464 | 0.458399 | 0.079 | 0.272 |
| KEGG_HEDGEHOG_SIGNALING_PATHWAY | 56 | 1.445 | 0.434513 | 0.076 | 0.288 |
| KEGG_NOD_LIKE_RECEPTOR_SIGNALING_PATHWAY | 62 | 1.441 | 0.427602 | 0.099 | 0.282 |
| KEGG_VIBRIO_CHOLERAE_INFECTION | 54 | 1.420 | 0.391865 | 0.074 | 0.298 |
| KEGG_GLIOMA | 65 | 1.411 | 0.397358 | 0.088 | 0.298 |
| KEGG_PANCREATIC_CANCER | 70 | 1.401 | 0.404685 | 0.111 | 0.303 |
| KEGG_AXON_GUIDANCE | 129 | 1.391 | 0.379621 | 0.094 | 0.305 |
| KEGG_INOSITOL_PHOSPHATE_METABOLISM | 54 | 1.385 | 0.430061 | 0.128 | 0.302 |
| KEGG_AMINOACYL_TRNA_BIOSYNTHESIS | 22 | 1.378 | 0.517645 | 0.162 | 0.300 |
| KEGG_N_GLYCAN_BIOSYNTHESIS | 46 | 1.367 | 0.444909 | 0.128 | 0.305 |
| KEGG_PRIMARY_IMMUNODEFICIENCY | 35 | -2.102 | -0.805314 | 0.000 | 0.015 |
| KEGG_INTESTINAL_IMMUNE_NETWORK_FOR_IGA_PRODUCTION | 46 | -2.047 | -0.760872 | 0.000 | 0.016 |
| KEGG_AUTOIMMUNE_THYROID_DISEASE | 50 | -2.021 | -0.720466 | 0.002 | 0.018 |

| | | | | | |
|---|---|---|---|---|---|
| KEGG_ASTHMA | 28 | -1.964 | -0.792507 | 0.002 | 0.028 |
| KEGG_ARACHIDONIC_ACID_METABOLISM | 58 | -1.916 | -0.542208 | 0.002 | 0.040 |
| KEGG_ALLOGRAFT_REJECTION | 35 | -1.885 | -0.807991 | 0.004 | 0.046 |
| KEGG_SYSTEMIC_LUPUS_ERYTHEMATOSUS | 55 | -1.813 | -0.671447 | 0.026 | 0.080 |
| KEGG_CELL_ADHESION_MOLECULES_CAMS | 131 | -1.797 | -0.547984 | 0.012 | 0.080 |
| KEGG_TYPE_I_DIABETES_MELLITUS | 41 | -1.783 | -0.693100 | 0.028 | 0.081 |
| KEGG_HEMATOPOIETIC_CELL_LINEAGE | 85 | -1.761 | -0.568026 | 0.024 | 0.088 |
| KEGG_NATURAL_KILLER_CELL_MEDIATED_CYTOTOXICITY | 132 | -1.733 | -0.512719 | 0.016 | 0.103 |
| KEGG_GRAFT_VERSUS_HOST_DISEASE | 37 | -1.708 | -0.731792 | 0.047 | 0.115 |
| KEGG_LEISHMANIA_INFECTION | 70 | -1.676 | -0.553619 | 0.054 | 0.138 |
| KEGG_FATTY_ACID_METABOLISM | 42 | -1.668 | -0.531167 | 0.024 | 0.135 |
| KEGG_CYTOKINE_CYTOKINE_RECEPTOR_INTERACTION | 264 | -1.655 | -0.446398 | 0.033 | 0.139 |
| KEGG_PARKINSONS_DISEASE | 127 | -1.650 | -0.547933 | 0.053 | 0.134 |
| KEGG_ANTIGEN_PROCESSING_AND_PRESENTATION | 81 | -1.648 | -0.565163 | 0.064 | 0.127 |
| KEGG_ALZHEIMERS_DISEASE | 165 | -1.641 | -0.458914 | 0.043 | 0.126 |
| KEGG_T_CELL_RECEPTOR_SIGNALING_PATHWAY | 108 | -1.633 | -0.493802 | 0.042 | 0.126 |
| KEGG_PEROXISOME | 78 | -1.621 | -0.446708 | 0.030 | 0.129 |
| KEGG_TRYPTOPHAN_METABOLISM | 39 | -1.612 | -0.505357 | 0.029 | 0.130 |
| KEGG_CARDIAC_MUSCLE_CONTRACTION | 78 | -1.610 | -0.502809 | 0.028 | 0.125 |
| KEGG_OXIDATIVE_PHOSPHORYLATION | 131 | -1.607 | -0.542387 | 0.070 | 0.123 |
| KEGG_B_CELL_RECEPTOR_SIGNALING_PATHWAY | 75 | -1.594 | -0.491494 | 0.048 | 0.128 |
| KEGG_LINOLEIC_ACID_METABOLISM | 29 | -1.593 | -0.533346 | 0.045 | 0.124 |
| KEGG_BUTANOATE_METABOLISM | 34 | -1.590 | -0.503871 | 0.038 | 0.120 |
| KEGG_ALDOSTERONE_REGULATED_SODIUM_REABSORPTION | 42 | -1.586 | -0.471743 | 0.050 | 0.119 |
| KEGG_CHEMOKINE_SIGNALING_PATHWAY | 188 | -1.564 | -0.442850 | 0.051 | 0.131 |
| KEGG_VALINE_LEUCINE_AND_ISOLEUCINE_DEGRADATION | 43 | -1.555 | -0.524273 | 0.056 | 0.134 |
| KEGG_FC_EPSILON_RI_SIGNALING_PATHWAY | 79 | -1.553 | -0.432609 | 0.036 | 0.132 |
